# Adenosine-induced ventricular fibrillation in a patient with supraventricular tachycardia

**DOI:** 10.5339/qmj.2021.52

**Published:** 2021-10-07

**Authors:** Shu Yu Lee, Sohil Pothiawala, Chong Meng Seet

**Affiliations:** ^1^Department of Emergency Medicine, Woodlands Health Campus, National Healthcare Group, Singapore E-mail: shu_yu_lee@whc.sg; ^2^Department of Emergency Medicine, Sengkang General Hospital, Singhealth Services, Singapore

**Keywords:** adenosine, ventricular fibrillation, supraventricular tachycardia

## Abstract

Adenosine is frequently used for paroxysmal supraventricular tachycardia (PSVT) treatment in the emergency department (ED). Atrial and ventricular pro-arrhythmic effects of adenosine were described in the literature, but ventricular fibrillation (VF) secondary to adenosine administration was rarely reported (with an incidence of < 1%). Reported herein is the first case of a 72-year-old female patient who developed VF hemodynamic collapse after an intravenous administration of adenosine for PSVT treatment. She had no known pre-excitation or accessory pathway, nor any underlying structural heart disease or prolonged QT syndrome. Raising awareness of this potential life-threatening pro-arrhythmic effect of adenosine is important, given its frequent use for PSVT treatment in the ED.

## Introduction

Paroxysmal supraventricular tachycardia (PSVT) refers to any tachydysrhythmia arising from above the level of the Bundle of His and encompasses atrioventricular re-entrant tachycardia (AVRT) and atrioventricular nodal re-entry tachycardia (AVNRT). AVNRT is the most common cause of palpitations in patients with a structurally normal heart, which involves an anatomical re-entry circuit within the AV node. Vagal maneuvers aid in conversion to sinus rhythm, and the modified Valsalva maneuver effectiveness was well documented in both the pre-hospital and hospital settings compared to that of the traditional method.^1^ Adenosine is the mainstay of treatment,^2^ but other agents, such as calcium channel blockers, beta-blockers, and amiodarone are also used. Adenosine blocks the AV nodal conduction and increases the AV nodal refractory period, thereby interrupting the re-entry pathway through the AV node, resulting in AVNRT termination.^3^

## Case report

A 72-year-old female patient presented to the emergency department (ED) after a primary care physician referral for tachycardia, who saw her with complaints of epigastric discomfort, vomiting, and loss of appetite for the past 2 days. An electrocardiogram (ECG) at the clinic revealed a narrow complex tachycardia of 169 beats/min. She was admitted for ischemic stroke 6 months earlier and was on aspirin. During that admission, PSVT developed but was successfully treated with intravenous (IV) adenosine. She was reviewed by the cardiologist, and 2D echocardiography was performed, which did not reveal any underlying structural heart abnormalities. In addition, an electrophysiological study confirmed the underlying etiology of PSVT as AVNRT.

**Figure 1.**
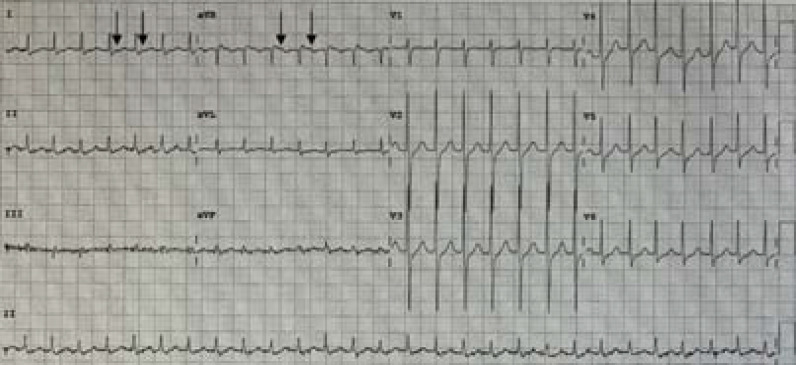
Electrocardiogram (ECG) showing a narrow complex tachycardia with retrograde P waves in leads I and aVR (arrows) and absent P waves in other leads.

On arrival, she was alert with a temperature of 37.3°C; heart rate of 170 beats/min; respiratory rate of 25 cycles/min; blood pressure of 112/81 mm/Hg; and oxygen saturation of 97% on room air. Her physical examination was normal. The ECG revealed a narrow complex tachycardia suggestive of AVNRT due to the presence of retrograde conduction of P waves in leads I and aVR (Figure 1). Previous ECG documented in electronic health records did not show any signs of pre-excitation for AVRT from an accessory pathway or prolonged QTc syndrome. She was observed on continuous cardiac rhythm monitoring throughout her ED stay.

Vagal maneuver (Valsalva) was attempted without success. A carotid sinus massage was not performed as the patient had a stroke history. IV adenosine at 6 mg was given for pharmacological conversion. Shortly after IV adenosine administration, the patient became unresponsive and developed a generalized tonic-clonic (GTC) seizure. Pulse was absent, and the cardiac monitor revealed ventricular fibrillation (VF). Cardiopulmonary resuscitation was immediately started together with the preparation for defibrillation. However, just before defibrillation, the seizure spontaneously aborted and she had a palpable pulse with a return of spontaneous circulation (ROSC). The whole episode of seizure and VF lasted for approximately 8–10 s. The cardiac monitor revealed a normal sinus rhythm conversion and the patient spontaneously regained consciousness. Repeat vital signs were stable with a heart rate of 72 beats/min; blood pressure of 102/62 mm/Hg, and oxygen saturation of 99% on room air.

**Figure 2.**
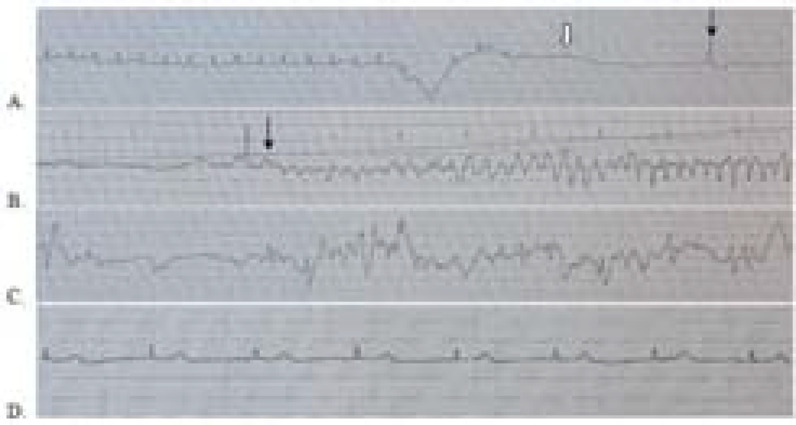
Rhythm strips showing (A) the adenosine-induced atrioventricular block interspersed with non-conducted P wave (white arrow) and ventricular escape beat (black arrow), (B) onset of ventricular fibrillation (black arrow), (C) spontaneous termination of ventricular fibrillation, and (D) reversion to sinus rhythm.

**Figure 3.**
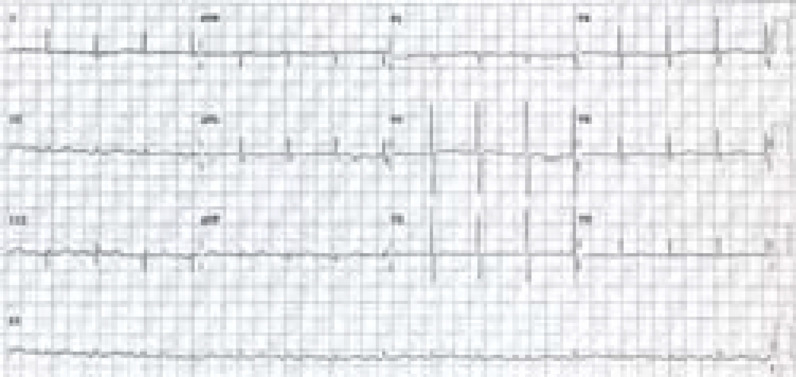
Repeat ECG showing normal sinus rhythm post adenosine administration.

A rhythm strip (Figure 2) review recorded during the IV adenosine administration revealed an initial suppression of the AV conduction lasting for approximately 6 s interspersed, with non-conducted P waves and ventricular escape beats (Figure 2A), in which the last escape beat triggered the onset of VF (Figure 2B), which lasted for approximately 8 s. The VF duration corresponded to the GTC seizure duration, indicating a convulsive syncope secondary to VF resulting in seizure. The patient does not have any history of epilepsy. This was followed by spontaneous VF termination and continued AV conduction suppression with a period of aberrantly conducted supraventricular impulses (Figure 2C) before sinus rhythm reversion (Figure 2D). A repeat ECG post adenosine administration did not reveal any pre-excitation features (Figure 3).

Blood investigations were done in the ED upon patient arrival before adenosine administration. These included a complete blood count with hematocrit level, serum electrolytes, and thyroid function test, which all revealed normal results. Coupled with her normal physical examination without signs of dehydration and ECG that was not suggestive of sinus tachycardia, the possibility of dehydration as a contributory factor to the tachycardia was ruled out. The patient was admitted to the High Dependency Unit (HDU) under cardiology. During admission, her serial cardiac enzymes were normal. Her symptoms of epigastric discomfort and vomiting were attributed to gastritis. She had another episode of SVT in the HDU, which was successfully converted with IV diltiazem. Myocardial perfusion imaging was negative for ischemia, and the transthoracic echocardiography revealed a normal ejection fraction without regional wall motion abnormalities. An electrophysiological study was not performed during this admission due to her advanced age and other co-morbidities. She was discharged stable after a few days and was started on long-term oral diltiazem.

## Discussion

Adenosine is indicated for PSVT (AVNRT and orthodromic AVRT) conversion to sinus rhythm. Antidromic AVRT presents as a wide complex QRS tachycardia due to antegrade conduction occurring via the accessory pathway and retrograde conduction via the AV node. The rate is usually 200–300 beats/min and confirmation diagnosis is difficult in the acute setting. Adenosine administration is dangerous in patients with SVT involving an accessory pathway, including Wolff-Parkinson-White syndrome, thus should be avoided.^4^

Adenosine was studied to effectively cardiovert a subset of idiopathic ventricular tachycardia (VT), particularly the right ventricular outflow tract tachycardia, which is typically catecholamine-induced.^5^ Adenosine antagonizes the cyclic adenosine monophosphate-mediated catecholamine stimulation of the ventricular muscle, which explains this form of VT as adenosine-sensitive. However, in patients with known structural heart disease presenting with regular wide complex tachycardia, where re-entry circuit is the most common mechanism for VT manifestation due to abnormal myocardial scarring from prior ischemia or infarction, adenosine is contraindicated as it worsens the arrhythmia and precipitates VF.^6^

Atrial and ventricular pro-arrhythmic effects of adenosine were described. A paradoxically increased tachycardia, when used in AVNRT, was reported. These patients were found to have dual AV node pathways where adenosine preferentially blocks the fast antegrade pathway, allowing a re-entry circuit to develop. In addition, adenosine was found to increase the ventricular rate when used in orthodromic AVRT.^7^ In these patients, a critical antegrade AV nodal conduction prolongation allowed a retrograde atrial activation via an accessory pathway, causing the emergence of a reciprocating tachycardia. Adenosine-induced ventricular arrhythmias were commonly reported in structurally normal hearts and normal QT intervals.^8^ These include premature ventricular contractions (PVC) and non-sustained VT, which are clinically insignificant, requiring no intervention. Significant ventricular arrhythmias such as paroxysmal ventricular tachycardia (PVT), torsades de pointes, and VF are more rarely encountered, causing serious adverse outcomes that require advanced life support measures. Non-sustained and sustained torsades de pointes that require cardioversion occurred after adenosine administration for SVT.^9^ Another case of adenosine-induced VF resulting in a hemodynamic collapse that required defibrillation was reported,^10^ but the presenting ECG was later reviewed to be atrial fibrillation with rapid ventricular response rather than SVT. Our case is the first report about VF with hemodynamic collapse secondary to adenosine treatment for AVNRT, in the absence of structural heart disease or prolonged QT syndrome.

Several studies outlined the mechanisms behind adenosine-induced atrial and ventricular arrhythmias. Adenosine shortens the effective refractory period in atrial myocytes and predisposes to atrial arrhythmias through increased spatial dispersion of refractoriness.^11^ A case of adenosine-induced VF was observed as a pause-dependent phenomenon,^12^ whereby atrioventricular conduction block that resulted in a long sinus pause after an adenosine administration is accompanied by a compensatory response, provoking the re-initiation of tachyarrhythmias in an attempt to maintain cardiac output. In addition, underlying acquired or congenital long QT syndrome precipitate life-threatening bradycardia-induced PVT/VF,^13^ whereby the slowing of ventricular rate during the adenosine administration results in progressive QT prolongation, which reflects prolonged repolarization and gives rise to early-after depolarization (EAD). These EADs manifest as PVCs and are known as the R on T phenomenon when occurring in the preceding T wave, causing torsades de pointes. Moreover, adenosine causes a reflex increased circulating catecholamine levels and sympathetic nerve traffic by sympathetic stimulation in the carotid body chemoreceptors.^14^ Catecholamine release causes increased ventricular automaticity as demonstrated in the A2 adenosine receptor in the ventricular myocytes. These mechanisms are responsible for the pro-excitatory effects of adenosine administration in VT/VF development.

In our patient, a low dose of adenosine at 6 mg induced a malignant ventricular arrhythmia, a rare idiosyncratic response especially in the absence of prolonged QT syndrome and structural heart diseases. Furthermore, the patient had a PSVT history, which was successfully converted to sinus rhythm without complications. This further demonstrates the inherent pro-arrhythmic potential of adenosine when used. The possible mechanism resulting in VF in our case would likely be that of a pause-dependent phenomenon followed by increased catecholamine levels and sympathetic stimulation; provoking the initiation of tachyarrhythmia. The short duration of hemodynamic collapse and ROSC reflects the transient effect on the atrioventricular node and rapid adenosine inactivation in the human plasma by uptake into the erythrocytes and endothelial cells.

## Conclusion

Adenosine remains a safe and effective agent for PSVT treatment in the ED. Clinicians are well aware of the side effects, such as bronchospasm, chest pain, and bradycardia, but its potential to induce life-threatening tachyarrhythmias are rare, albeit transient in nature owing to its short duration of action. Therefore, during adenosine administration, the emergency physician must bear in mind the possibility of cardiovascular collapse from malignant arrhythmias. All patients must be placed on continuous cardiac monitoring during cardioversion and prompt access to resuscitation and defibrillation equipment must be ensured.

### Source(s) of support/Funding

Nil

### Acknowledgment

Nil

### Presentation at the meeting

Nil

### Conflict of interest

All the authors have no potential conflict of interest

### Author's contribution list

Shu Yu Lee is responsible for manuscript preparation, literature search, and drafting of the initial manuscript. Sohil Pothiawala and Chong Meng Seet reviewed and edited the manuscript, followed by final approval of the manuscript for submission.

All authors have read and approved the final draft of the manuscript.
